# Effect of simethicone for the management of early abdominal distension after laparoscopic cholecystectomy: a multicenter retrospective propensity score matching study

**DOI:** 10.1186/s12893-024-02460-w

**Published:** 2024-05-29

**Authors:** Yi Zhu, Jinjie Li, Ji Gao, Dousheng Bai, Zhengping Yu, Shengjie Jin, Jianfei Chen, Shuang Li, Ping Jiang, Zhong Ge, Minchao Liu, Chuandong Sun, Yongjie Su, Yubin Zhang, Yong Zhang

**Affiliations:** 1 Hepatobiliary and Pancreatic Surgery, The Second Affiliated Hospital, Zhejiang University School of Medicine, Hangzhou, 310009 China; 2 Hepatobiliary and Pancreatic Surgery, The 1st Affiliated Hospital of Wenzhou Medical University, Wenzhou, 325015 China; 3 Hepatobiliary Surgery, Jiangsu Province Hospital, The First Affiliated Hospital with Nanjing Medical University, Nanjing, 210029 China; 4 Hepatobiliary and Pancreatic Surgery, Northern Jiangsu People’s Hospital, Yangzhou, 225001 China; 5 Hepatobiliary Oncology Surgery Department, Beijing Shijitan Hospital, Beijing, 100000 China; 6 General Surgery Department, The First Affiliated Hospital of Dalian Medical University, Dalian, 116011 China; 7 Hepatobiliary and Pancreatic Surgery, Zhongnan Hospital of Wuhan University, Wuhan, 430000 China; 8 Hepatobiliary and Pancreatic Surgery, Qingdao Municipal Hospital, Qingdao, 266000 China; 9 Hepatobiliary Hernia Surgery, Zhangzhou Affiliated Hospital of Fujian Medical University, Zhangzhou, 363000 China; 10 Hepatobiliary and Pancreatic Surgery, The Affiliated Hospital of Qingdao University, Qingdao, 266000 China; 11 Hepatobiliary Surgery, Zhongshan Hospital Xiamen University, Xiamen, 361000 China; 12 Hepatobiliary Surgery, Shijiazhuang People’s Hospital, Shijiazhuang, 050000 China; 13 Hepatobiliary and Pancreatic Surgery, Union Hospital, Tongji Medical College, Huazhong University of Science and Technology, No. 1277 Jiefang Avenue, Wuhan, 430022 China

**Keywords:** Early abdominal distension, Enhanced recovery after surgery (ERAS), Laparoscopic cholecystectomy (LC), Simethicone

## Abstract

**Objective:**

To investigate whether simethicone expediates the remission of abdominal distension after laparoscopic cholecystectomy (LC).

**Methods:**

This retrospective study involved LC patients who either received perioperative simethicone treatment or not. Propensity score matching (PSM) was employed to minimize bias. The primary endpoint was the remission rate of abdominal distension within 24 h after LC. Univariable and multivariable logistic regression analyses were conducted to identify independent risk factors affecting the early remission of abdominal distension after LC. Subsequently, a prediction model was established and validated.

**Results:**

A total of 1,286 patients were divided into simethicone (*n* = 811) and non-simethicone groups (*n* = 475) as 2:1 PSM. The patients receiving simethicone had better remission rates of abdominal distension at both 24 h and 48 h after LC (49.2% vs. 34.7%, 83.9% vs. 74.8%, respectively), along with shorter time to the first flatus (14.6 ± 11.1 h vs. 17.2 ± 9.1 h, *P* < 0.001) compared to those without. Multiple logistic regression identified gallstone (OR = 0.33, *P* = 0.001), cholecystic polyp (OR = 0.53, *P* = 0.050), preoperative abdominal distention (OR = 0.63, *P* = 0.002) and simethicone use (OR = 1.89, *P* < 0.001) as independent factors contributing to the early remission of abdominal distension following LC. The prognosis model developed for predicting remission rates of abdominal distension within 24 h after LC yielded an area under the curve of 0.643 and internal validation a value of 0.644.

**Conclusions:**

Simethicone administration significantly enhanced the early remission of post-LC abdominal distension, particularly for patients who had gallstones, cholecystic polyp, prolonged anesthesia or preoperative abdominal distention.

**Trial registration:**

ChiCTR2200064964 (24/10/2022).

**Supplementary Information:**

The online version contains supplementary material available at 10.1186/s12893-024-02460-w.

## Introduction

Laparoscopic cholecystectomy (LC) is generally preferred to open gallbladder resection due to its minimally invasive nature together with a faster postoperative recovery and better cosmesis [1]. Even though LC has changed the current paradigm for managing gallbladder diseases, postoperative gastrointestinal dysfunction (POGD), especially abdominal distension has been reported to be one of the most frequent complications, which directly affects the postoperative recovery as well as the quality of life of patients [2].

Post-LC POGD commonly results from extensive intraoperative manipulation of the gastrointestinal tract, prolongation of general anesthesia, excessive residual CO_2_ gas in the abdominal cavity, longer tissue shock and other systemic comorbidities. This complication presents with a range of symptoms, including abdominal distension, delayed defecation, intestinal obstruction, gastrointestinal bleeding, enterogenic infection and even multiple organ dysfunctions [3–5]. Hence, the early diagnosis, prevention and treatment of POGD are essential components of Enhanced Recovery After Surgery (ERAS) protocols [4, 6, 7].

According to previous studies, abdominal distension and other POGD symptoms have been commonly managed with either dimethicone with pancreatin, calcium antagonists or other drugs for symptomatic control [2]. Nevertheless, the experimental results reported in the literature to date have indicated that the defoaming effect of simethicone was greater than that of dimethicone [8, 9], suggesting its potential for the treatment of postoperative abdominal distension.

Simethicone, as a stable non-ionic surfactant, can rupture air bubbles in the digestive tract by reducing their surface tension and preventing the development of mucus-surrounded gas pockets throughout the intestine [10]. Since the pharmacological functions of simethicone depend on its physical properties, it can be excreted in its original form from the gastrointestinal tract without absorption into the bloodstream following oral administration, thereby ensuring a favorable safety profile [11]. Initially approved for use by the United States Food and Drug Administration in 1952 [12, 13], simethicone has demonstrated efficacy in improving global symptoms and bloating in patients with irritable bowel syndrome (IBS), as evidenced by a randomized, placebo-controlled trial involving the addition of simethicone and pinaverium bromide to the therapy regimen [14].

Although simethicone has been clinically used for bowel preparation before colonoscopy, there is no strong evidence on whether it can be recommended for the management of abdominal distention after LC. Therefore, the aims of the present study were to investigate the efficacy and safety of simethicone in alleviating early postoperative abdominal distension after LC and to ascertain its role in ERAS protocols after LC.

## Patients and methods

### Patients

The inclusion criteria for patient enrollment encompassed individuals who were: (1) aged between 18 and 70 years; (2) scheduled for elective LC between June 2021 and December 2022; and (3) possessed satisfactory medical records. Exclusion criteria were for patients who had: (1) received emergency LC, single port LC, combined endoscopic retrograde cholangiopancreatography, procedure conversion or combined drainage placement; (2) abnormal liver and kidney functions (abnormal liver function defined as aspartate aminotransferase, alanine aminotransferase or total bilirubin levels ≥ 2 times the upper reference limit; abnormal renal function indicated by a creatinine clearance rate < normal); (3) severe cardiac and pulmonary insufficiency; (4) a history of severe gastrointestinal disease (such as irritable bowel syndrome, constipation, etc.); (5) a previous abdominal surgery history; (6) neurological or mental illness or other psychological illnesses that disabled cooperation; (7) progressive malignant tumors or other serious consumptive diseases; (8) unstable acute and chronic diseases; (9) chemotherapy or radiotherapy or other treatments that may have affected the efficacy evaluation; (10) postoperative ICU admission due to severe intraoperative complications; (11) ongoing pregnancy and lactation; or (12) severe postoperative diarrhea.

This real-world multicenter retrospective study enrolled 1,520 patients who underwent LC in 34 tertiary hospitals across China from June 2021 to December 2022. The patients were stratified into simethicone treated (1,017) and non-simethicone treated (503) groups for comprehensive analysis based on the primary and secondary study objectives for this study. Patient data was retrieved from the electronic medical records system.

The effectiveness and safety of simethicone was assessed by the researchers through analysis of changes in clinical symptoms associated with the medication use before the study commencement. The study protocol was approved by the Medical Ethics Committee of Union Hospital, Tongji Medical College, Huazhong University of Science and Technology (Approval No. 2023 − 0124), and informed consent was obtained from all participating patients. In addition, the study was registered with the Chinese Clinical Trial Registry (ChiCTR) as ChiCTR2200064964 (registration date: 24/10/2022). All procedures and methods were conducted in accordance with relevant guidelines and regulations, and strictly followed the STROCSS 2021 guidelines [15].

### Outcomes and measurements

#### Primary endpoint

The primary endpoint of this study was the remission rate of abdominal distension within 24 h of LC, which was evaluated by determining the number of patients with abdominal distension 6 h post-LC but not 24 h post-LC, divided by the total number with abdominal distension within 6 h after the operation.

#### Secondary endpoints

The secondary endpoints included the following parameters: (1) time to first flatus; (2) remission rate of abdominal distension 48 h after the LC operation; (3) remission rate of abdominal distension during the first week after LC; (4) remission rate of abdominal distension during the second week after the operation; (5) incidence of abdominal distension 6 h after LC; (6) incidence of abdominal distension 24 h after LC; (7) frequency and duration of patients use of a postoperative analgesic pump; (8) duration of the first abdominal distension after the operation; (9) number of patients with first passage of flatus within 6 h after the operation; (10) recovery of bowel sounds 6 h after the operation; (11) number of patients with intraoperative gastrointestinal flatulence; (12) time of the first postoperative ambulation; and (13) the length of hospital stay.

#### Safety

Adverse events (AEs) attributed to simethicone administration were detailed as any unexpected medical condition that occurred after the patient received drug treatment, regardless of the existence of the causal relationship with the treatment agent. AE severity was graded using the Medical Dictionary for Regulatory Activities (MedDRA): grade 1 for mild AEs; grade 2 for moderate AEs; grade 3 for severe and undesirable AEs; grade 4 for life threatening or disabling AEs; and grade 5 for AEs resulting in death. Additionally, serious AEs (SAEs) included those that led to hospital admission, prolonged hospitalization, permanent/serious disability, precipitated organ dysfunction, and other serious medical events or death.

### Measurements

The remission of symptoms after simethicone administration were evaluated using a scoring system designed to track changes in objective symptoms at various time point, along with a rating of bowel sound improvement.

Although the focus of this study was on abdominal distention after LC, unified criteria for grading abdominal distension remain elusive. To address this issue, we implemented a modified verbal rating scale (a binary scale indicating presence or absence), derived from information extracted from inpatient or outpatient medical record systems [16] in the participating hospitals, to objectively assess the degree of abdominal distension post-LC surgery [6]. Clinical symptoms of abdominal fullness were scored as as follows: 0 indicated no symptoms of abdominal distention, 1 indicated the presence of symptoms of abdominal distention which included mild discomfort in the abdomen, noticeable abdominal discomfort, sustained abdominal distention, often accompanied by a sensation of nausea and vomiting, and severe sustained abdominal distention accompanied by bloating (Supplementary Table 1). Moreover, evaluation of postoperative abdominal distention was scheduled on the evening before the surgery, then at 6 h after surgery, on the morning of postoperative day 1 (24 h post-LC), day 2 (48 h post-LC), day 7 (1 week post-LC) and day 14 (2 weeks post-LC).

#### Medication and surgery

Preoperatively, selected patients were instructed to adhere to a regimen that prohibited a regular diet after midnight and a liquid diet 2 h before the induction of general anesthesia. Patients orally received simethicone emulsion at a dosage of 200 mg, diluted in 10–20 mL of water, once 24 h prior to the operation and again 6 h post-surgery. Subsequently, patients received 80 mg of simethicone, diluted in 10–20 mL of water, three times a day for 7 days, beginning 24 h after LC and continuing for 1 week after surgery.

LC procedures were performed under general anesthesia, with patients positioned supine. Pneumoperitoneum was maintained as a preset pressure of 12–14 mmHg. The three-port approach was the choice for the majority of patients, which involved insertion of a 10-mm trocar through the umbilical incision close to the umbilicus, a 10-mm port in the midline epigastrium circa 2 cm below the xiphoid process, and a 5-mm trocar in the right mid-clavicular line positioned about 2 cm from the costal margin. In cases requiring a four-port approach, an additional 5-mm trocar was inserted into the right hypochondrium on the anterior axillary line, 3 cm below the costal margin.

Each patient was then positioned in the reverse Trendelenburg position, with a left-down tilt. The gallbladder was dissected in the bottom-up fashion, ensuring complete exposure of Calot’s triangle anatomy, followed by identifying, as well as preserving, the common bile duct and then ligating the cystic duct and vessels before removing the gallbladder. An intraoperative cholangiogram was not mandated and minimal manipulation of the bowel was undertaken during the operation.

### Statistical methods

All statistical analyses were conducted using R4.2.1 software, with significance set at a two-sided *P*-value < 0.05. Descriptive statistics were used to analyze demographic information, baseline characteristics data, disease history and other general patient data. Continuous and categorical variables are presented as the median quartile and *n* (%), respectively. The Mann-Whitney U was employed for inter-group comparisons of continuous variables, and the chi-squared test was used to compare categorical variables. Multiple imputation methods were employed to fill in any missing data for the analyses.

Baseline demographics and clinical features, including age, gender, weight, gallbladder stones, cholecystitis, gallbladder polyps, other gallbladder diseases, pre-operative abdominal distension, intra-operative flatulence, hypertension, hyperlipidemia, diabetes, coronary heart disease, operation duration and anesthesia duration were all taken into account in the analyses. All primary and secondary endpoints were evaluated based on the intention-to-treat principle. Given the initial imbalance in baseline data between the groups, propensity score matching (PSM) was applied to minimize bias. Logistic regression was used to calculate propensity scores for each patient, enabling re-matching of individuals with similar scores at 2:1 for the simethicone and control groups. Following PSM, odd ratios (OR) and 95% confidence intervals (CI) were calculated to assess the effect of simethicone on postoperative abdominal distension.

Univariable and multivariable logistic regression analyses were used to establish prognostic factors. All variables in the univariable analysis were added to the logistic regression model to generate model 1; the meaningful variables in multivariate analysis were included in the logistic regression model to construct model 2; the stepwise regression method was adopted to choose the meaningful variables for model 3. Receiver operating characteristic (ROC) curves were generated for each model, with predictive ability assessed using the area under the ROC curve (AUC). A nomogram was constructed based on significant prognostic factors from model 3, allowing the prediction of abdominal distension remission 24 h after surgery. Internal validation of model 3 was performed using 1,000 bootstrap resamples [17], which were further evaluated by AUC, the Hosmer-Lemeshow goodness-of-fit test (HL test), and calibration plot and decision curve analysis (DCA).

### Sufficiency of sample size

This retrospective study enrolled a total of 1,520 patients, with 1,017 in the simethicone group and 503 in the control group, according to the inclusion and exclusion criteria (*vide supra*). Subsequent sample size calculations were performed to ensure adequacy. Given the absence of precise data in the existing literature, the primary endpoint of this study, the abdominal distension remission rate 24 h after elective LC-was set at 35% based on clinical experience. Assuming an 8% increase in the remission rate with simethicone treatment, the Z-test method was used to calculate the rate of two independent samples with a two-sided α of 0.05 and a power of 0.85 in the difference between the two groups. Allowing for approximately 10% of data dropout, the calculation reached a power of 0.81 for detecting differences in the 24-h postoperative abdominal distension remission rate between the two groups.

## Results

### Demographic covariables of patients before and after PSM adjustment

**Fig. 1:**
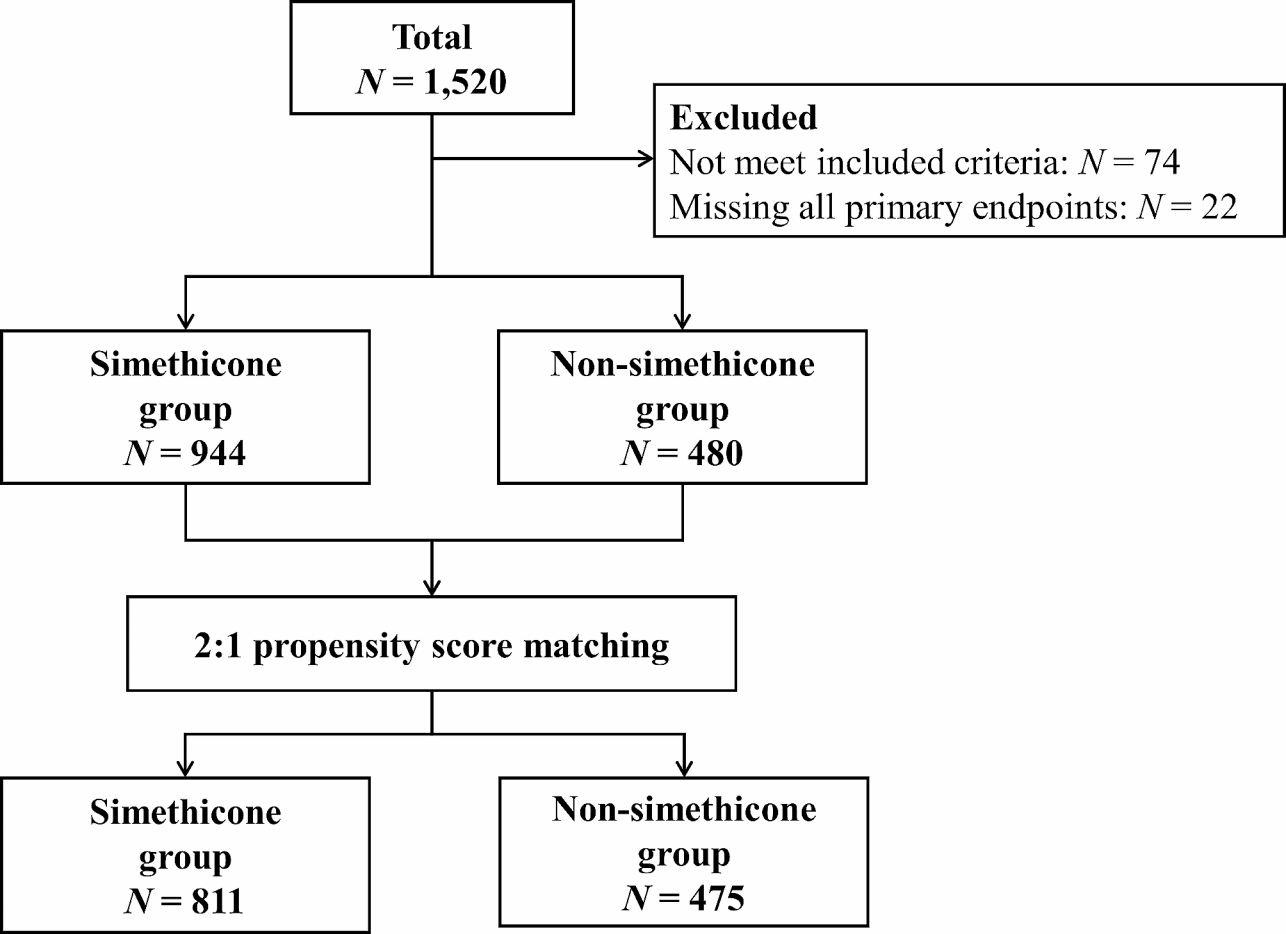
Flow chart of the selection of study patients before and after PSM. PSM, propensity score matching

A total of 1,424 patients were retrospectively selected from a pool of 1,520 patients treated in 34 medical centers and then assigned to either the simethicone group (*n* = 944) or the non-simethicone group (*n* = 480). Since significant differences existed in the variables of other gallbladder diseases, hyperlipidemia, operating time and duration of anesthesia (all *P* < 0.05) between the simethicone and non-simethicone groups (Table 1), PSM was performed to reduce confounding biases. Following 2:1 PSM, while a significant difference in the operation duration remained, the baseline covariables between the simethicone group (*n* = 811) and the non-simethicone group (*n* = 475) were balanced (Supplementary Fig. S1, Fig. 1 and Table S2).

**Table 1:** Demographic baseline and clinical features of the study cohort before and after PSM

	Before 2:1 PSM					After 2:1 PSM				
	Simethicone group (*n* = 944)	Non-simethicone group (*n* = 480)	*P*-value	Overall (*n* = 1,424)	SMD	Simethicone group (*n* = 811)	Non-simethicone group (*n* = 475)	*P*-value	Overall (*n* = 1,286)	SMD
Male	379 (40.1)	175 (36.5)	0.196	554 (38.9)	0.076	306 (37.7)	173 (36.4)	0.682	479 (37.2)	0.027
Age	51 (41, 59)	50 (39, 60)	0.497	51 (40, 59)	0.036	50 (40, 58)	50 (39, 60)	0.985	50 (40, 59)	0.002
Weight	63 (56, 70)	63 (55, 71)	0.644	63 (56, 70)	0.088	63 (57, 70)	63 (55, 71)	0.929	63 (56, 70)	0.056
Gallstones	821 (87.0)	409 (85.2)	0.404	1230 (86.4)	0.051	703 (86.7)	406 (85.5)	0.600	1109 (86.2)	0.035
Cholecystitis	460 (48.7)	251 (52.3)	0.224	711 (49.9)	0.071	421 (51.9)	250 (52.6)	0.848	671 (52.2)	0.014
Gallbladder polyps	109 (11.5)	68 (14.2)	0.183	177 (12.4)	0.078	99 (12.2)	66 (13.9)	0.431	165 (12.8)	0.050
Other gallbladder diseases	30 (3.2)	27 (5.6)	0.037	57 (4.0)	0.12	26 (3.2)	24 (5.1)	0.133	50 (3.9)	0.093
Preoperative abdominal distension	302 (32.0)	138 (28.8)	0.378	440 (30.9)	0.136	240 (29.6)	138 (29.1)	0.887	378 (29.4)	0.012
Hypertension	133 (14.1)	60 (12.5)	0.456	193 (13.6)	0.047	102 (12.6)	59 (12.4)	1.000	161 (12.5)	0.005
Hyperlipidemia	31 (3.3)	4 (0.8)	0.008	35 (2.5)	0.173	9 (1.1)	4 (0.8)	0.862	13 (1.0)	0.027
Coronary heart disease	11 (1.2)	4 (0.8)	0.760	58 (4.1)	0.033	7 (0.9)	4 (0.8)	1.000	11 (0.9)	0.002
Diabetes	44 (4.7)	14 (2.9)	0.152	15 (1.1)	0.091	23 (2.8)	14 (2.9)	1.000	37 (2.9)	0.007
Duration of surgery	50 (40, 70)	45 (30, 60)	< 0.001	50 (35, 67)	0.104	50 (37.5, 69)	45 (30, 60)	0.001	50 (35, 65)	0.047
Duration of anesthesia	75 (57.8, 95)	70 (50, 90)	< 0.001	73.5 (55, 90)	0.147	73 (55, 90)	70 (50, 90)	0.050	70 (55, 90)	0.068

Data are presented as a *number* (percentages) or median (Q1, Q3)

PSM, propensity score matching; SMD, standardized mean difference

### Primary endpoints before and after PSM

**Fig. 2:**
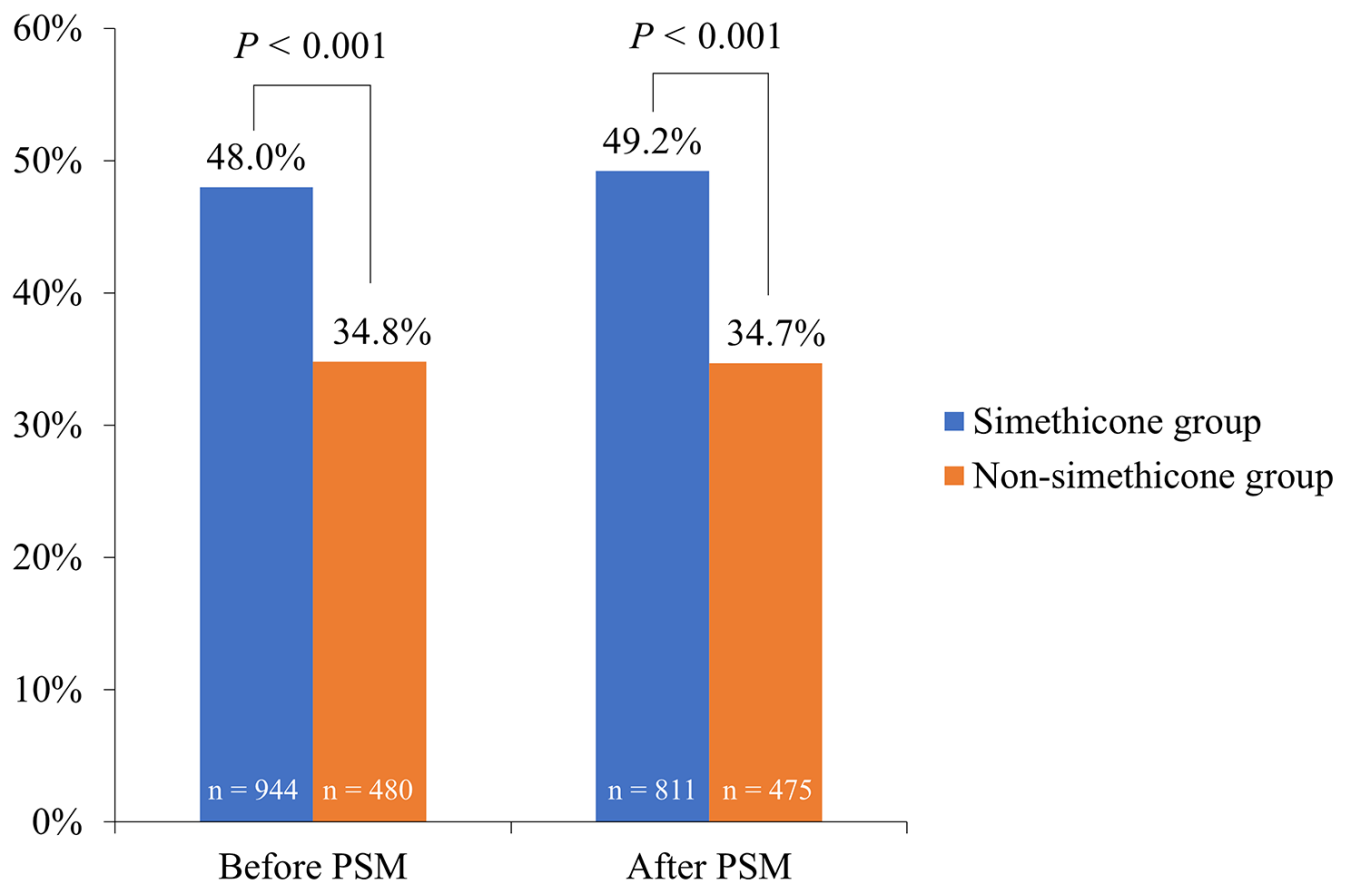
Remission rates of abdominal distension in the two groups 24 h after LC. LC, laparoscopic cholecystectomy

Before and after PSM adjustment, a comparison was made between the remission rates of abdominal distension 24 h after operation in both groups, demonstrating a significantly higher remission rate in the simethicone group compared to the non-simethicone group (48.0% vs. 34.8%, *P* < 0.001; 49.2% vs. 34.7%, *P* < 0.001) (Fig. 2).

### Secondary endpoints

**Fig. 3:**
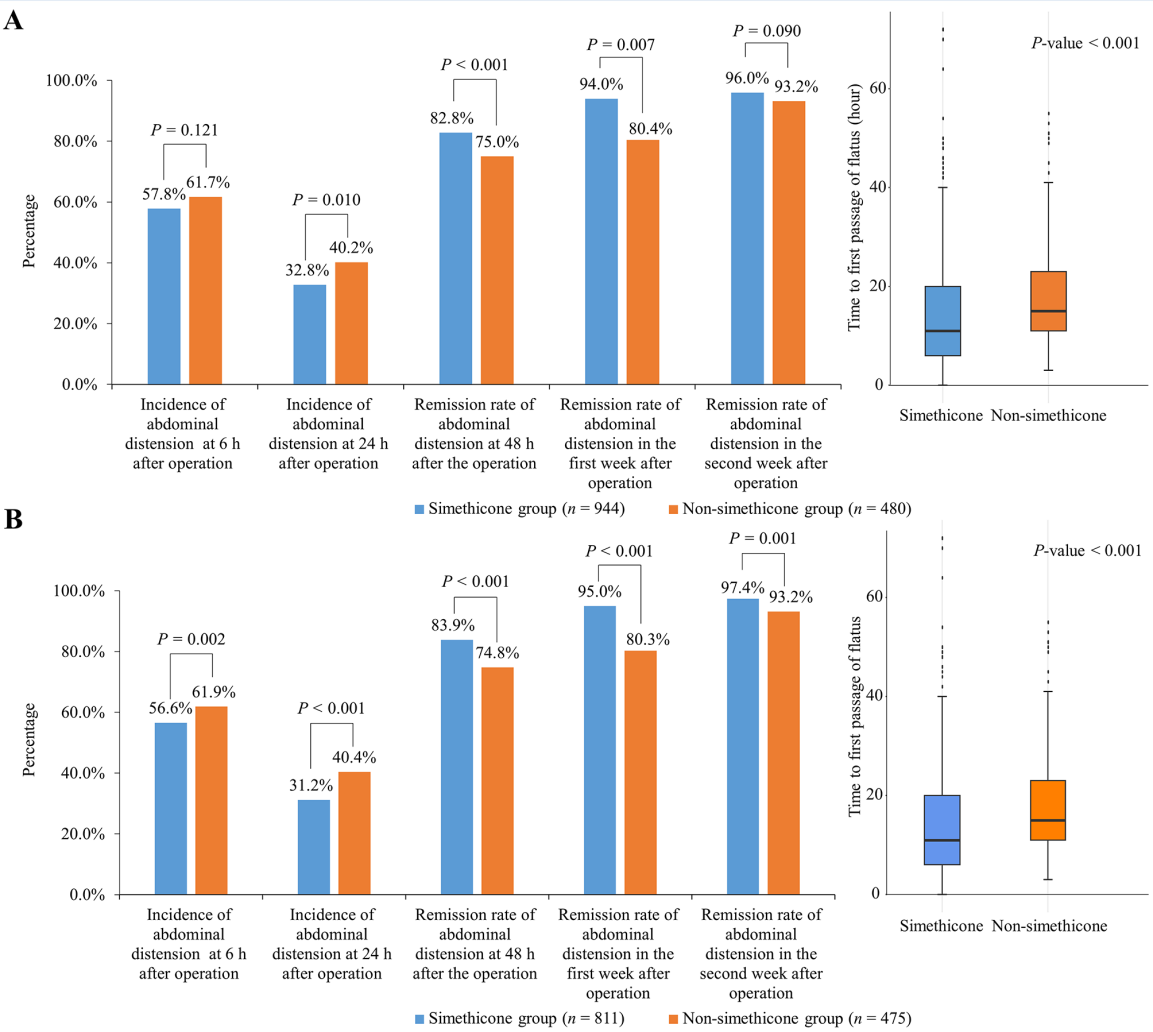
Secondary endpoints before (**A**) and after PSM (**B**) PSM, propensity score matching

Before and after PSM, significant differences were found in the incidence of abdominal distension 24 h post-operation (32.8% vs. 40.2%, *P* = 0.010; 31.2% vs. 40.4%, *P* < 0.001), remission rates of abdominal distension at 48 h post-operation (82.8% vs. 75.0%, *P* < 0.007; 83.9% vs. 74.8%, *P* < 0.001), 1 week post-operation (94.0% vs. 80.4%, *P* = 0.007; 95.0% vs. 80.3%, *P* < 0.001), and the time of the first anal exhaust post-operation (14.8 ± 10.9 vs. 17.5 ± 8.9, *P* < 0.001; 14.6 ± 11.1 vs. 17.2 ± 9.1, *P* < 0.001) between the two groups. However, significant differences were noticed only after PSM in the incidence of abdominal distension 6 h after LC and the remission rate of abdominal distension at 2 weeks after LC (Fig. 3).

To compare the difference in primary and secondary endpoint indicators between the simethicone group and the non-simethicone group before and after PSM, we conducted a univariable and multivariable logistic analyses. Before PSM, the OR for the remission rate of abdominal distension at 24 h in the simethicone group was 1.79 (95% CI: 1.31, 2.44, *P* < 0.001), whereas in the post-PSM group, the OR for the remission rate of abdominal distension at 24 h was 2.16 (95% CI: 1.56, 2.99, *P* < 0.001). However, concerning the incidence of abdominal distension at 6–24 h after LC, simethicone group did not show a significant advantage (Table 2).

**Table 2:** Unadjusted and adjusted primary and secondary endpoints in the pre- and post-PSM population groups

	Group		Unadjusted		Adjusted	
	Simethicone group	Non-simethicone group	*OR* (95% *CI*)	*P*-value	*OR* (95% *CI*)	*P*-value
**Pre-PSM**	*n* = 944	*n* = 480				
**Primary endpoint**						
Remission rate of abdominal distension at 24 h ^a^	263 (48.0%)	103 (34.8%)	1.73 (1.29, 2.32)	< 0.001	1.79 (1.31, 2.44)	< 0.001
**Secondary endpoints**						
Incidence of abdominal distension at 6 h ^b^	546 (57.8%)	296 (61.7%)	0.85 (0.68, 1.07)	0.165	0.82 (0.64, 1.05)	0.121
Incidence of abdominal distension at 24 h ^b^	310 (32.8%)	193 (40.2%)	0.73 (0.58, 0.91)	0.006	0.72 (0.57, 0.93)	0.010
Remission rate of abdominal distension at 48 h ^a^	452 (82.8%)	222 (75.0%)	1.60 (1.14, 2.26)	0.007	1.65 (1.15, 2.36)	0.007
Remission rate of abdominal distension in the 1st week after LC ^a^	513 (94.0%)	238 (80.4%)	3.79 (2.41, 5.97)	< 0.001	2.94 (2.42, 6.43)	< 0.001
Remission rate of abdominal distension in the 2nd week after LC ^a^	524 (96.0%)	276 (93.2%)	1.73 (0.93, 3.22)	0.086	1.78 (0.91, 3.50)	0.090
**Post-PSM**	*n* = 811	*n* = 475				
**Primary endpoint**						
Remission rate of abdominal distension at 24 h after LC ^a^	226 (49.2%)	102 (34.7%)	1.83 (1.35, 2.47)	< 0.001	2.16 (1.56, 2.99)	< 0.001
**Secondary endpoints**						
Incidence of abdominal distension at 6 h after LC ^b^	459 (56.6%)	294 (61.9%)	0.80 (0.64, 1.01)	0.063	0.66 (0.51, 0.86)	0.002
Incidence of abdominal distension at 24 h after LC ^b^	253 (31.2%)	192 (40.4%)	0.67 (0.53, 0.85)	0.001	0.55 (0.43, 0.71)	< 0.001
Remission rate of abdominal distension at 48 h after LC ^a^	385 (83.9%)	220 (74.8%)	1.75 (1.22, 2.51)	0.003	2.21 (1.50, 3.27)	< 0.001
Remission rate of abdominal distension in the first week LC ^a^	436 (95.0%)	236 (80.3%)	4.66 (2.80, 7.74)	< 0.001	7.09 (4.01, 12.53)	< 0.001
Remission rate of abdominal distension in the second week after LC ^a^	447 (97.4%)	274 (93.2%)	2.72 (1.31, 5.65)	0.007	4.12 (1.87, 9.10)	0.001

CI, confidence interval; LC, laparoscopic cholecystectomy; OR, odds ratio; PSM, propensity score matching

^a^ Abdominal distension remission rates at 24 h, 48 h, 1-week and 2-weeks after LC were calculated as that the number of patients who did not experience abdominal distension at 24 h, 48 h, 1-week and 2-weeks after LC divided by the number of patients who experienced abdominal distension 6 h after LC.

^b^ The incidence of abdominal distension at 6 h and 24 h after operation were calculated as that the total number of patients with abdominal distension at 6 h and 24 h after LC divided by the total number of patients

### Independent factors affecting the remission of abdominal distension 24 h after surgery in the PSM population by univariable and multivariable analyses

The univariable analysis demonstrated that gallstone (OR = 0.51, 95% CI: 0.35–0.76, *P* = 0.001), preoperative abdominal distention (OR = 0.65, 95% CI: 0.50–0.86, *P* = 0.003), anesthesia duration (OR = 0.99, 95% CI: 0.99–1.00, *P* < 0.001), operating time (OR = 0.99, 95% CI: 0.98–1.00, *P* < 0.001), simethicone use (OR = 1.73, 95% CI: 1.29–2.32, *P* < 0.001) were 5 significant factors associated with the development of abdominal distension 24 h after selective LC. However, the multivariable analysis revealed that gallstone (OR = 0.33, 95% CI: 0.18–0.62, *P* = 0.001), cholecystic polyp (OR = 0.53, 95% CI: 0.28–1.00, *P* = 0.050) preoperative abdominal distention (OR = 0.63, 95% CI: 0.47–0.85, *P* = 0.002), simethicone use (OR = 1.89, 95% CI: 1.39–2.58, *P* < 0.001) were 4 significant factors related to the development of abdominal distension 24 h after selective LC (Table 3).

**Table 3:** Univariate and multivariate analyses of the factors affecting remission of abdominal distension 24 h after LC

Factors	Univariable analysis		Multivariable analysis	
	OR (95% CI)	*P*-value	OR (95% CI)	*P*-value
Gender, male	0.91 (0.69, 1.20)	0.507		
Age	1.00 (0.99, 1.01)	0.843		
Weight	1.00 (0.99, 1.01)	0.678		
Gallstone	0.51 (0.35, 0.76)	0.001	0.33 (0.18, 0.62)	0.001
Cholecystitis	0.98 (0.74, 1.28)	0.856		
Cholecystic polyp	1.17 (0.78, 1.76)	0.451	0.53 (0.28, 1.00)	0.050
Other gallbladder disease	1.74 (0.84, 3.64)	0.138		
Preoperative abdominal distention	0.65 (0.50, 0.86)	0.003	0.63 (0.47, 0.85)	0.002
Hypertensive	0.86 (0.58, 1.28)	0.455		
Hyperlipidemia	1.85 (0.90, 3.83)	0.097		
Coronary heart disease	0.49 (0.13, 1.84)	0.289		
Simethicone use	1.73 (1.29, 2.32)	< 0.001	1.89 (1.39, 2.58)	< 0.001
Diabetic mellitus	1.48 (0.77, 2.84)	0.240		
Operating time	0.99 (0.98, 1.00)	< 0.001		
Anesthesia duration	0.99 (0.99, 1.00)	< 0.001		

Note: Multivariate indexes with significant differences were included to analyze the predicted value

CI, confidence interval; LC, laparoscopic cholecystectomy; OR, odds ratio

**Fig. 4:**
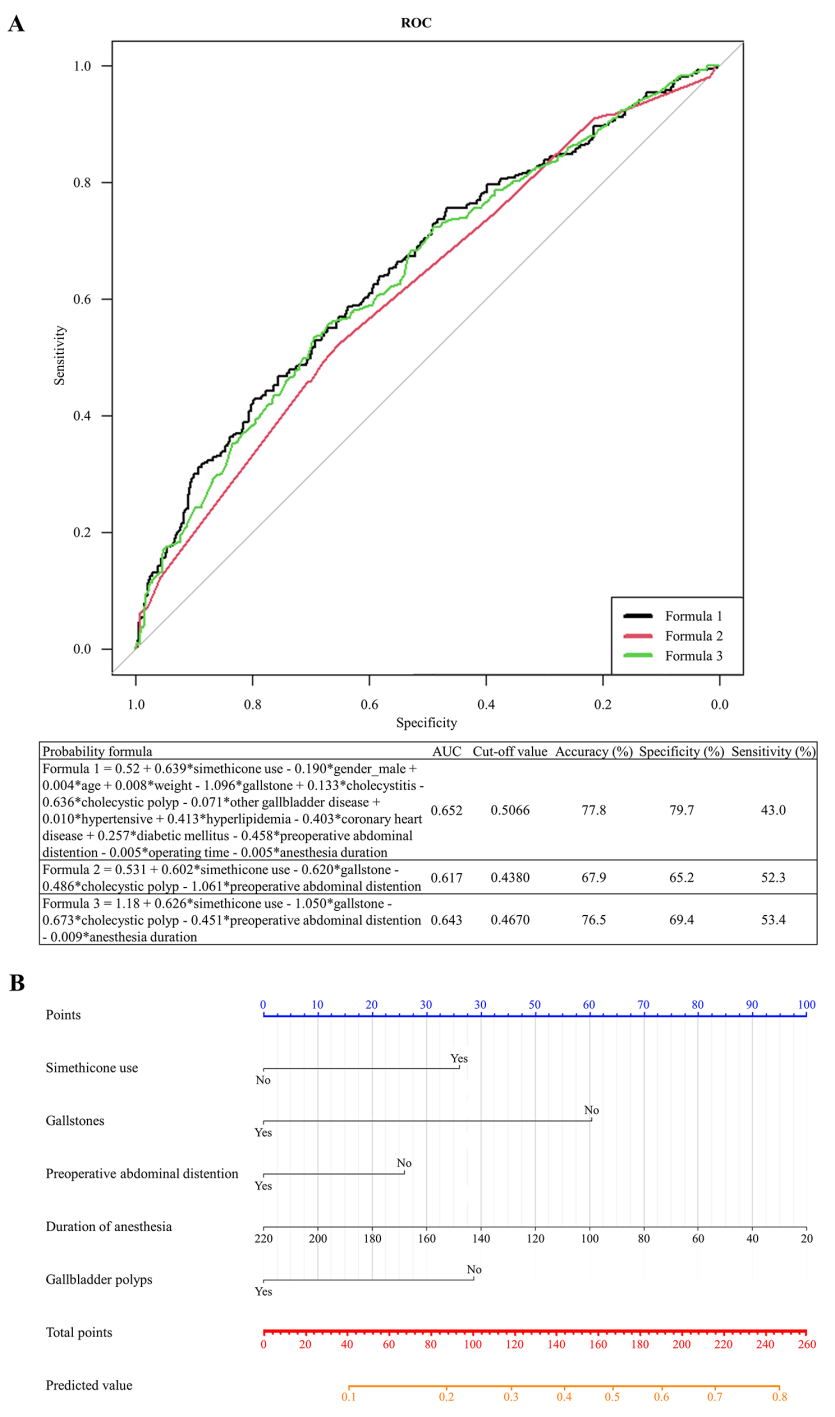
The prediction probability of the development of abdominal distension 24 h after LC for the pre-PSM population. (**A**) probability value by ROC analysis, (**B**) nomogram prediction. PSM, propensity score matching; ROC, receiver-operating characteristic

Furthermore, employing the stepwise regression method, significant factors associated with the development of abdominal distension 24 h after selective LC were identified as the presence of gallstones, cholecystic polyp, preoperative abdominal distention, simethicone treatment and anesthesia duration. In addition, ROC curves (Fig. 4A) were plotted to establish 3 predictive models to assess the efficacy of the simethicone treatment in achieving the remission rate of abdominal distension 24 h after the operation. The AUCs of models 1, 2 and 3 derived from three respective formulas were 0.652, 0.617 and 0.643, of which formula 3 (*vide supra*) was selected based on its superior sensitivity, specificity and accuracy (Fig. 4A).

Subsequently, a nomogram was constructed based on the relative weights of each prognostic factor (preoperative medication, gallstones, preoperative abdominal distention, anesthesia time, and gallbladder polyps) as indicated in formula 3. Each predictive factor was delineated on individual rows, with varying points assigned corresponding to their magnitude. The cumulative point axis at the end of the nomogram enables an assessment of overall points, where a higher total indicates a more substantial benefit in achieving the remission rate of abdominal distension 24 h after LC (Fig. 4B). Furthermore, the ROC curve of the nomogram model was plotted, revealing an AUC of 0.643 (95% CI: 0.605–0.681) for the original model. Internal validation of the model was performed using Bootstrap resampling with 1,000 iterations, yielding an AUC of 0.644 (95% CI: 0.607–0.678). Meanwhile, the HL test for the model yielded a χ^2^ value of 9.067, with a corresponding *P*-value of 0.337. Calibration curve demonstrated excellent agreement between predicted and observed rates of abdominal distention remission 24 h post-surgery (Supplementary Fig. S2A). Significantly, the DCA results depicted that at high-risk thresholds ranging from 25 to 67%, the “logistic model” curve (i.e., the nomogram model) outperformed the two extreme scenarios (None-line and All-line) (Supplementary Fig. S2B).

### Safety endpoints

The incidence of patients vomiting in the simethicone group (*n* = 179, 19.0%) was less than in the non-simethicone group (*n* = 138, 28.8%), statistically significant differences (*P* ≤ 0.001) across various observation times (Table 4, Supplementary Table S3). However, upon meticulous case review, the researchers concluded that postoperative vomiting was not directly correlated with the use of simethicone but rather postoperative abdominal distention. Besides, simethicone did not elicit the symptoms of abdominal pain, diarrhea and other AEs. No reports of SAEs were documented throughout the duration of this study.

**Table 4:** Occurrence of adverse events in the simethicone and control group

Before PSM	Number of patients with vomiting (incidence)	Total number of vomiting events
Simethicone group (*n* = 944)	179 (19.0%)	374
Non-simethicone group (*n* = 480)	138 (28.8%)	479
Chi-squared analysis	*P* < 0.001	

PSM, propensity score matching

### Other endpoints

**Fig. 5:**
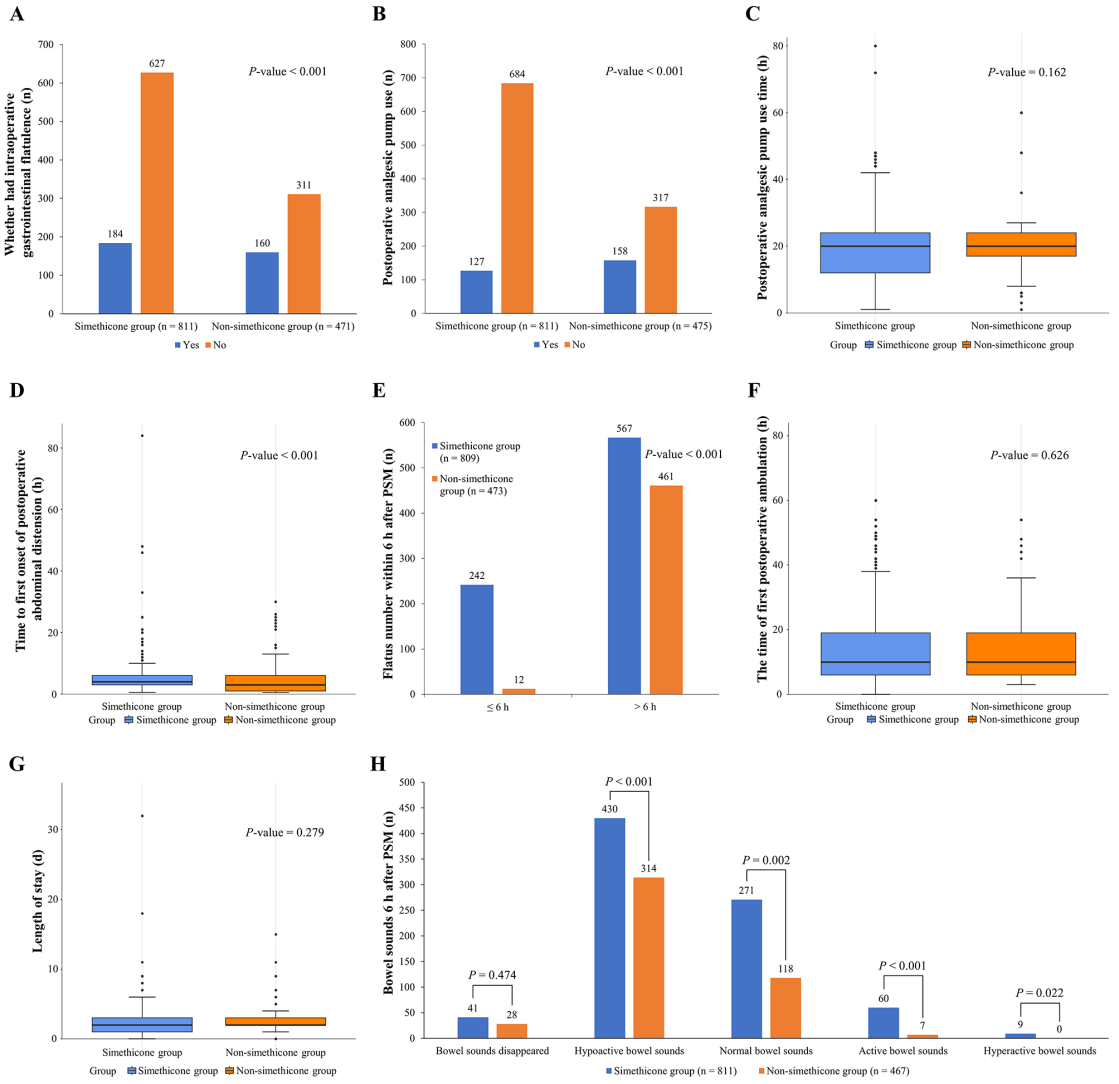
Comparison of other endpoints for the simethicone and control groups. (**A**) Occurrence of intra-operative gastrointestinal flatulence, (**B**) Rate of post-operative analgesic use, (**C**) Duration of post-operative analgesic use, (**D**) Time to the initial onset of abdominal distension, (**E**) Number of flatus within 6 h after PSM, (**F**) Time to the first post-operative ambulation, (**G**) Length of hospital stay, (**H**) Bowel sounds 6 h after PSM. PSM, propensity score matching

The additional endpoints included the number of intraoperative flatulence events after PSM, the number of postoperative analgesic pump users after PSM, the time of postoperative analgesic pump use after PSM, the time to the first abdominal distension after PSM, the number of bowel sounds 6 h after PSM, the time to postoperative ambulation after PSM, the length of hospital stay before PSM and the time to exhaust within 6 h after PSM, all of which were compared between the two cohorts. The analysis revealed that simethicone use conferred advantages across most of these parameters (Fig. 5 and Supplementary Table S3).

## Discussion

This multicenter retrospective PSM study evaluated the efficacy of simethicone in alleviating early post-operative abdominal distension following elective LC and yielded several significant findings. First, simethicone elicited a significant reduction in postoperative abdominal distension compared to the non-simethicone group, both in the immediate and delayed phases after LC. Second, perioperative use of simethicone significantly accelerated the onset of the first flatus after the surgery. Third, simethicone possessed a favorable safety profile, without the occurrence of high-grade AEs. Finally, simethicone conferred benefits to patients with gallbladder stones, a prolonged duration of operation and general anesthesia, and preoperative abdominal distention.

As the first study to investigate the effects of simethicone on POGD in patients who underwent LC, the findings align well with earlier trials evaluating the efficacy of simethicone in managing POGD in gynecological surgery patients. These demonstrated simethicone’s significant reductions of abdominal distension, abdominal pain, ileus, number of rectal treatments (suppositories, enemas), opioid use, as well as the time to the first spontaneous flatus and stool [9]. Since both LC and gynecologic surgery involve minimal bowel manipulation, simethicone’s efficacy in managing POGD can be corroborated in patients undergoing either of these procedures. It is worth noting that our study used higher doses and a longer duration of simethicone administration compared to previous trials [9, 18–20]. This extended regimen may account for the significant benefits observed for simethicone use in the present study.

Prior to the laparoscopic era, postoperative POGD was a common complication following major abdominal surgeries, contributing to significant morbidity, prolonged hospital stays and increased healthcare costs [21]. The widespread adoption of laparoscopy in diverse abdominal surgeries has markedly reduced bowel manipulation in non-gastrointestinal procedures, resulting in a more benign and transient manifestation of POGD in patients undergoing laparoscopic hepatectomy, cholecystectomy and gynecological procedures. The present study presents compelling evidence that simethicone use for one week significantly reduced abdominal distension from the first.

24 h up to the second week after surgery, underscoring the importance of considering simethicone in POGD management for ERAS protocols.

Further univariable and multivariable logistic regression analyses were conducted to investigate potential contributing factors to the development of abdominal distention [22]. Logistic regression revealed that the presence of covariables, namely gallstones, cholecystitis, gallbladder polyps, pre-operative abdominal distension, intra-operative flatulence and pre-operative medication, all played significant roles in not only decreasing the early and late remission of abdominal distension after LC, but also increasing the incidence of abdominal distension and delaying bowel motility after the operation. Furthermore, ROC analysis was performed to propose three predictive models based on various variables, which include gender, age, body weight, gallstones, cholecystitis, cholecystic polyp, other gallbladder disease, preoperative abdominal distention, hypertensive, hyperlipidemia, coronary heart disease, diabetic mellitus, operation time, anesthesia duration and preoperative simethicone treatment, to predict the possibility of development of abdominal distension after LC. After internal validation, model 3 was chosen to identify perioperative simethicone use, gallstone, cholecystitis, preoperative abdominal distension and anesthesia duration for sensitive variables that could predict the development of post-LC abdominal distension compared with the other 2 models. The results suggested that the development of post-LC abdominal distension was multi-factorial in nature and therefore its prevention and treatment should be more inclusive.

We acknowledge that the present study had several limitations. First, even though the robust sample size made our study reach appropriate outcomes with adequate statistical power to detect significances in the remission of abdominal distension following simethicone use or not, the retrospective nature of the study did lower the evidence level. Second, variation in the administered doses of simethicone to some patients due to various AEs introduced errors in the statistical analyses. Third, potential recall bias could have influenced the results, as medical records served as the primary data source, some of which were indeed deficient with some important information missing. Fourth, this study faced difficulties in assessing abdominal distension due to the lack of a universally accepted scale. To address this, our team worked with clinical experts to develop scoring criteria prior to the start of the study. Based on common clinical symptoms and physical examinations, we developed an abdominal distension scoring method. However, its subjective nature and limited validation limit its clinical applicability. In addition, the reliance on historical medical records in this retrospective study may compromise the accuracy and comparability of the data. Therefore, study conclusions should be interpreted with caution. Future research should prioritize the development and validation of a standardized, objective rating scale to improve the clinical assessment of abdominal distension and ensure the comparability and generalizability of research results.

## Conclusion

This multicenter retrospective study, employing propensity score matching, is the first investigation into the efficacy and safety of simethicone administration in ameliorating abdominal distension after LC. The results conclusively demonstrated that simethicone effectively relieved early abdominal distension and facilitated the recovery of bowel function subsequent to LC, particularly in patients presenting with gallbladder stones, preoperative abdominal distension and prolonged durations of general anesthesia or surgical operations.

### Electronic supplementary material

Below is the link to the electronic supplementary material.

Supplementary Material 1

## Data Availability

The datasets used and/or analysed during the current study are available from the corresponding author on reasonable request.

## Abbreviations

AE: Adverse event
AUC: Area under the ROC curve
ERAS: Enhanced recovery after surgery
IBS: Irritable bowel syndrome
LC: Laparoscopic cholecystectomy
MedDRA: Medical Dictionary for Regulatory Activities
OR: Odds ratio
POGD: Postoperative gastrointestinal dysfunction
PSM: Propensity score matching
ROC: Receiver operating characteristic
SAE: Serious adverse event
