# Supplementary material for: Effect of simethicone for the management of early abdominal distension after laparoscopic cholecystectomy: a multicenter retrospective propensity score matching study
Source: BMC Surg. 2024 May 29;24:170. doi: 10.1186/s12893-024-02460-w (PMC11134702; doi:10.1186/s12893-024-02460-w)
Supplement: Supplementary file 1 — Supplementary Material 1 [file 12893_2024_2460_MOESM1_ESM.docx]

**Supplementary Table S1** Abdominal distension scoring table

| Abdominal Distension（Bloating） | |
| --- | --- |
| Absence | Presence |
| 0 | 1 |

**Supplementary Table S2** Incidence of vomiting in the two groups at different times

| Times | 0 | 1 | 2 | 3 | 4 | 5 | 6 | 7 | 8 | 9 | 10 | 13 |  |
| --- | --- | --- | --- | --- | --- | --- | --- | --- | --- | --- | --- | --- | --- |
| Simethicone group (*n* = 944) | 765 (81.0%) | 93 (9.9%) | 37 (3.9%) | 20 (2.1%) | 9 (1.0%) | 15 (1.6%) | 2 (0.2%) | 2 (0.2%) | 0 (0.0%) | 0 (0.0%) | 1 (0.1%) | 0 (0.00%) | *P* ≤ 0.001 |
| Non-simethicone group (*n* = 480) | 342 (71.2%) | 36 (7.5%) | 24 (5.0%) | 26 (5.4%) | 10 (2.1%) | 18 (3.8%) | 10 (2.1%) | 1 (0.2%) | 6 (1.2%) | 1 (0.2%) | 5 (1.0%) | 1 (0.2%) |  |

**Supplementary Table S3** Comparison of other endpoints for the simethicone and non-simethicone groups

| Variable |  | Simethicone | Non-simethicone | *P*-value |
| --- | --- | --- | --- | --- |
| Whether had intraoperative gastrointestinal flatulence (*n*) | Yes | 184 (22.7%) | 160 (34.0%) | < 0.001 |
|  | No | 627 (77.3%) | 311 (66.0%) |  |
| Postoperative analgesic pump use (*n*) | Yes | 127 (15.7%) | 158 (33.3%) | < 0.001 |
|  | No | 684 (84.3%) | 317 (66.7%) |  |
| Postoperative analgesic pump use time (h) | Minimum | 1.00 | 1.00 | 0.162 |
|  | Upper quartile | 11.75 | 16.60 |  |
|  | Median | 19.50 | 20.31 |  |
|  | Lower quartile | 24.48 | 24.05 |  |
|  | Maximum | 80.00 | 60.00 |  |
| Time to first onset of postoperative abdominal distension (h) | Minimum | 0.50 | 0.50 | < 0.001 |
|  | Upper quartile | 3.06 | 1.51 |  |
|  | Median | 4.35 | 3.51 |  |
|  | Lower quartile | 5.86 | 5.93 |  |
|  | Maximum | 48.00 | 30.00 |  |
| Flatus number within 6 h after PSM (*n*) | ≤ 6 h | 242 (29.9%) | 12 (2.5%) | < 0.001 |
|  | > 6 h | 567 (70.1%) | 461 (97.5%) |  |
| Bowel sounds 6 h after PSM (*n*) | Disappeared | 41 (5.1%) | 28 (6.0%) | 0.474 |
|  | Hypoactive | 430 (53.0%) | 314 (67.2%) | < 0.001 |
|  | Normal | 271 (33.4%) | 118 (25.3%) | 0.002 |
|  | Active | 60 (7.4%) | 7 (1.5%) | < 0.001 |
|  | Hyperactive | 9 (1.1%) | 0 (0.0%) | 0.022 |
| The time of first postoperative ambulation (h) | Minimum | 0 | 3.00 | 0.626 |
|  | Upper quartile | 6.02 | 6.33 |  |
|  | Median | 10.20 | 9.90 |  |
|  | Lower quartile | 18.78 | 18.89 |  |
|  | Maximum | 60.00 | 54.00 |  |
| Length of stay (d) | Minimum | 0 | 0 | 0.279 |
|  | Upper quartile | 1 | 2 |  |
|  | Median | 2 | 2 |  |
|  | Lower quartile | 3 | 3 |  |
|  | Maximum | 32 | 15 |  |

PSM, propensity score matching

**
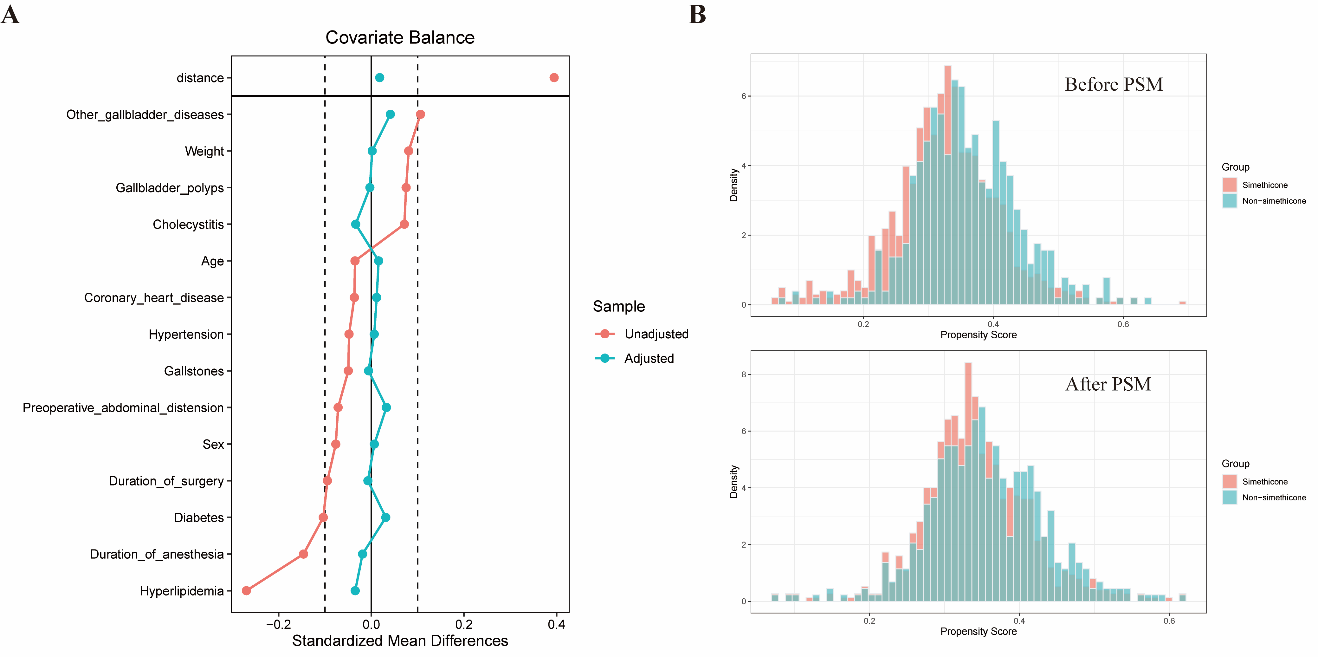
**

**Supplementary Fig. S1** Propensity score matching: balancing covariates (A) and sample distributions balance of “distance” (B)


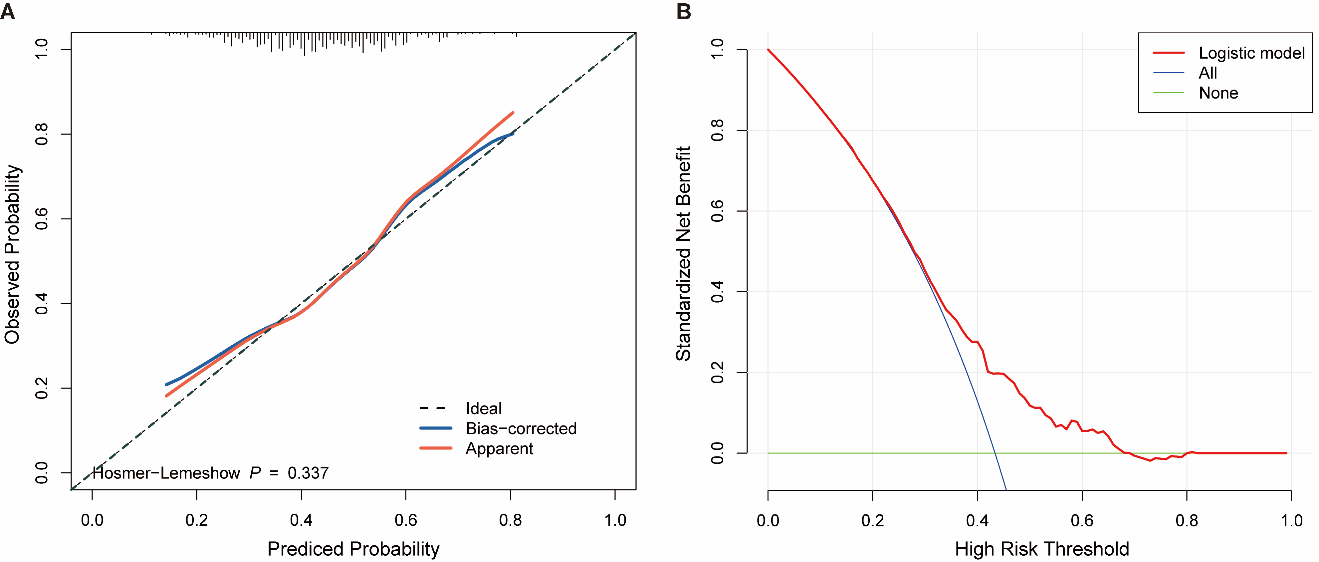


**Supplementary Figure S2.** Validation of the nomogram model.

(A) Calibration curve. (B) DCA curve.
